# Effects of Standard Humic Materials on Relative Bioavailability of NDL-PCBs in Juvenile Swine

**DOI:** 10.1371/journal.pone.0115759

**Published:** 2014-12-30

**Authors:** Matthieu Delannoy, Jessica Schwarz, Agnès Fournier, Guido Rychen, Cyril Feidt

**Affiliations:** 1 UR AFPA, Université de Lorraine, Vandœuvre-lès-Nancy, France; 2 UR AFPA, INRA USC 340, Vandœuvre-lès-Nancy, France; National Taiwan University, Taiwan

## Abstract

Young children with their hand-to-mouth activity may be exposed to contaminated soils. However few studies assessing exposure of organic compounds sequestrated in soil were realized. The present study explores the impact of different organic matters on retention of NDL-PCBs during digestive processes using commercial humic substances in a close digestive model of children: the piglet. Six artificial soils were used. One standard soil, devoid of organic matter, and five amended versions of this standard soil with either fulvic acid, humic acid, *Sphagnum* peat, activated carbon or a mix of *Sphagnum* peat and activated carbon (95∶5) (SPAC) were prepared. In order to compare the different treatments, we use spiked oil and negative control animals. Forty male piglets were randomly distributed in 7 contaminated and one control groups (n  = 5 for each group). During 10 days, the piglets were fed artificial soil or a corn oil spiked with 19 200 ng of Aroclor 1254 per g of dry matter (6 000 ng.g^−1^ of NDL-PCBs) to achieve an exposure dose of 1 200 ng NDL-PCBs.Kg^−1^ of body weight per day. NDL-PCBs in adipose tissue were analyzed by GC-MS. Fulvic acid reduced slightly the bioavailability of NDL-PCBs compared to oil. Humic acid and *Sphagnum* peat reduced it significantly higher whereas activated carbon reduced the most. Piglets exposed to soil containing both activated carbon and *Shagnum* peat exhibited a lower reduction than soil with only activated carbon. Therefore, treatment groups are ordered by decreasing value of relative bioavailability as following: oil ≥ fulvic acid>*Sphagnum* peat ≥ *Sphagnum* peat and activated carbon ≥ Humic acid>>activated carbon. This suggests competition between *Sphagnum* peat and activated carbon. The present study highlights that quality of organic matter does have a significant effect on bioavailability of sequestrated organic compounds.

## Introduction

Numerous epidemiological studies assessing neuropsychological impact of developmental exposure to polychlorinated biphenyls (PCBs) have linked neurobehavioral impairments with pre- or early postnatal exposure. Several cohort studies in children (at 7 months of age and at 4 years of age) related PCB exposure to a possible deficit of memory [1]. In rats, following a perinatal exposure, similar results were obtained [2]–[4], even at environmentally low doses [5]. Although toxicity pathways are not accurately known, some studies emphasize the major role of non-dioxin like PCB (NDL-PCBs) congeners [6], [7]. Numerous former industrial sites are known to be contaminated by those NDL-PCBs [8], [9].

This makes them almost ubiquitous in the environment, mainly in soil. Children are particularly susceptible to incidentally ingest those soil as they may exhibit hand-to-mouth activities. Thus, assessing exposure to pollutants from contaminated soils is an important issue in terms of safety management. Because digestive processes are known to be species dependent, the choice of an appropriate animal model is of critical importance for this exposure assessment. Indeed, interspecies variability constitute an emerging concern for organic compounds (OC) [10] since gastrointestinal absorption differs, as also reported for metallic ions [11], [12]. In order to achieve a quantity of PCBs relative to body weight under experimental conditions comparable to contaminated soil ingested by toddlers, the juvenile swine appears to be a valuable model. With a relative similarity of physiology, growth and absorptive mechanisms than humans, swine model is more and more used to study oral bioavailability of pollutants in ingested soil [10], [11], [13].

Thoroughly understanding the processes and mechanisms governing sequestration and mobility of Persistent Organic Pollutants (POPs) in soil during digestion is an important issue to address. As the 6 NDL-PCBs exhibit a wide range of lipophilic and stability properties, as well as an affinity for organic carbon, they are a challenging group to study. Indeed, their fate in the digestive system seems related to chemical and physical properties of the tested contaminant and its interaction and partition among the different constituents of the soil. In this perspective organic matter (OM) is commonly considered as the most important fraction limiting bioavailability of POPs in soil [14], [15]. In addition, the soil humic fraction has been previously reported to considerably retain NDL-PCBs [16]. Humic material has empirically been divided in three different fractions which could be obtained by acid-base extractions: 1/fulvic acid (FA) consists in a soluble fraction at any pH; 2/humic acid (HA) in a fraction soluble in base solutions but precipitating at pH<2; and 3/humin which is a fraction insoluble in an aqueous solution at any pH [16]. Because there is a lack of information on the mechanisms driving retention of POPs in each of the humic phases along the digestive processes, the impact of soil sequestration on NDL-PCBs bioavailability is not totally understood.

In a pilot study we focused on two contrasted organic matters opposed by their condensation degrees. Fulvic acid and activated carbon, were used to highlight the importance of differences in bioavailability induced by the type of OM [17]. This pilot study was performed and revealed that FA exhibited no retention of NDL-PCBs during digestive processes whereas 1% of activated carbon (AC) achieved almost complete sequestration of NDL-PCBs [17].

As natural soils presented a wide range of different qualities, the present study was conducted to assess the effects of different standardized OMs on the relative bioavailability (RBA) of NDL-PCBs. The oral RBA was assessed using juvenile swine by feeding them artificial soils containing PCBs and 1% of organic carbon brought by one humic substance. In addition to humic standards, one complex mixture of OM and the effect of competition between different sources of OM were tested.

## Materials and Methods

### 1 Experimental design

**Figure 1:**
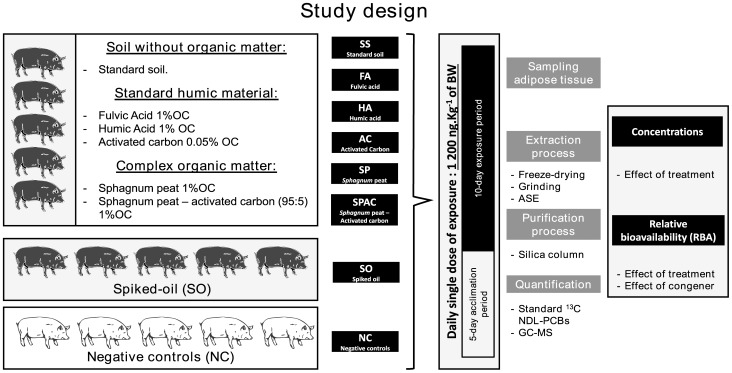
Animal management and experimental design of the study.

The experimental design to assess the impact of different OM qualities on relative bioavailability (RBA) of NDL-PCB is shown in Fig. 1. Eight groups of piglets were fed one of the eight experimental treatments for 10 days. RBA was estimated using concentrations of NDL-PCBs in adipose tissue of artificial soil-fed and corn oil-fed animals [17], [18].

Five swine were randomly assigned to each treatment group exposed to one of the eight exposure matrices (*cf* Table 1). The group of negative control (NC) swine was fed a non-contaminated standard soil (n  = 5) and all animals were sacrificed at the end of the contamination period (day 10). During the exposure period (day 1 to day 10) piglets were administered daily a moistened dough ball with either the spiked oil or the experimental soil. The exposure dose was set to 1 200 ng of NDL-PCBs per kg of body weight (BW) (corresponding to 3 840 ng of Aroclor 1254 in standard solution per kg of BW). To reach this exposure dose, the body weight of piglets was checked every two days in order to adapt the amount of each matrix introduced in dough ball (200±10 mg dry matter (DM) of oil or soil per Kg of piglet’s BW). Comparison between oil-fed and soil-fed animals groups was realized in order to assess exposure to spiked soil with OM in terms of exposure *via* the alimentary one. Similar methodology was previously used [19]–[21]. The present study provides RBA estimates using a single dose comprised in a linearity-tested range of doses [17].

**Table 1:** Composition of the different artificial soils and treatment of the experiment.

	Corn Oil	Sand	Kaolin	Fulvic acid	Humic acid	*Sphagnum* peat moss	Activated carbon	Aroclor1254	Time of maturation
	(Sigma-aldrich, StLouis, USA)	See Sand (Carl RothGmbH, Karlsruhe,Germany)	(Sigma-aldrich,St Louis, USA)	Swanee River FAstandard I (IHSS,St Paul, USA)	(Sigma-aldrich,St Louis, USA)	(Sphagnum peat mossfrom baltic countries,Verve, France)	Activated charcoal 242276(Sigma-aldrich,St Louis, USA)	Concentration(µg.g^−1^ of DM)(Sigma-aldrich, Supelco)	in days
Spiked corn oil (SO)	100%	–	–	–	–	–	–	19.2	–
Standard soil (SS)	–	77.80%	22.20%	–	–	–	–	19.2	21
Fulvic acid (FA)	–	76.20%	21.90%	1.92%	–	–	–	19.2	21
Sphagnum peat (SP)	–	76.00%	21.70%	–	–	2.33%	–	19.2	21
Humic Acid (HA)	–	75.80%	21.70%	–	2.56%	–	–	19.2	21
Sphagum peat andactivated carbon (SPAC)	–	75.80%	21.70%	–	–	2.38%	0.05%	19.2	21
Activated carbon (AC)	–	77.70%	22.20%	–	–	–	0.05%	19.2	21
Negative controls (NC)	–	77.80%	22.20%	–	–	–	–	–	21

### 2 Animals, ethics and housing

Crossbred (Pietrain x Youna) castrated male swine (7.6±1.4, mean±SE), weaned at 28 days of age were used. A 5-day acclimation period was realized prior to the start of the contamination. Housing and food management were previously described [17]. Shortly, animals were housed individually in stainless-steel cages with wire floors in the animal facility of UR AFPA (Université de Lorraine, Vandœuvre-les-Nancy France). Temperature was kept to 26–27°C. Every 2 days, BW of piglets was monitored. Feed (Porcelet Super VE 25 Kg, Lorial, Laxou, France) was provided daily at 4.5% of BW. Access to feed were provided one hour after administration of treatment. Daily ingestion was estimated by subtracting leftovers from provided ration. Water was provided *ad libitum* by nipple waterers throughout the entire study. This study was carried out in strict accordance with the recommendations in the Guide for the Care and Use of Laboratory Animals of the French Ministry of Agriculture for Animal Research (MAAR) and European Council Directive (European directive 2010/63/EU). Furthermore, the protocol was approved by the “Comité d’Éthique Lorrain en Matière d’Expérimentation Animale (CELMEA)” (Permit Number: 00344.01, delivered by the secretariat of Ministère de l’Education Superieure et de la Recherche, France).

### 3 Dosing material and method of exposure to NDL-PCBs

#### 3.1 Exposure matrices

Spiked oil (SO) was prepared as a reference matrix using corn oil (Sigma Aldrich, St Louis, USA). In addition, eight artificial soils were prepared as described in Table 1 according to the Organization for Economic Cooperation and Development (OECD) guideline 207 [22]. The standard soil (SS) contained sand and kaolin only (Sigma-Aldrich). This soil fulfilled composition and pH of an OECD artificial soil except for the *Sphagnum* peat, which was not introduced to avoid sorption competition between the different OMs. In addition to the SS, two different sets of artificial soils were used. The first set of three soils contained simple and well characterized organic matter: SS plus fulvic acid (FA) (Suwannee River II, IHSS, St. Paul, USA), SS plus humic acid (HA) (Humic acid, Sigma Aldrich) and SS plus activated carbon (Activated charcoal, Darco −100 mesh particle size, Sigma Aldrich). The second set containing two soils with complex organic matter: SS plus *Sphagnum* peat (SP) and SS plus a mix of *Sphagnum* peat and activated carbon (95∶5) (SPAC). Each organic matter was introduced at the same mass concentration of 1% of organic carbon as this soil characteristic may impact on the capacity of retention of NDL-PCBs. One exception has been realized for the soil containing activated carbon (AC) which was introduced at 0.05%. This difference was made in order to compare retention of the mix of peat and activated carbon with a soil containing the same concentration of activated carbon as the only source of OM. Chemicals used, spiking techniques and preparation of soils are described in Table 1 and were realized as previously detailed [17].

#### 3.2 Spiking technique

All the seven matrices (oil and soils) were spiked with 19 200 ng of Aroclor 1 254 (Sigma Aldrich, Supelco) per g of DM. Resulting amounts corresponded to 6 000 ng of NDL-PCBs per g of DM of spiked oil. This single dose is comprised in a tested range of doses [17]. Because linearity was prior validated, concentrations in adipose tissue are proportional to absorbed dose of NDL-PCBs [10], [17], [20]. After spiking soil and oil matrices, solvent traces were evaporated under an extractor hood overnight. All soils were stored at 20°C in amber glass vials during three weeks of maturation prior to the first day of exposure. Oil was stored at 4°C in amber glass vials.

### 4 Sampling and analysis of biological samples

After 10 days of exposure piglets, electronarcosis was induced prior immediate exsanguination. Pericaudal adipose tissue was collected. Adipose tissue as used in this study is known to be the most important tissue for distribution and accumulation of NDL-PCBs in mammals [2]. All analytical steps from preparation of samples (freeze-drying, milling) to chemical analyses were realized as described before [17]. In short, these analyses involved three successive steps (Fig. 1). Assisted solvent extraction from freeze-dried and milled samples of adipose tissue was performed (ASE 350, Dionex, Sunnyvale, USA) and ^13^C-labelled NDL-PCBs from LGC Standard (Molsheim, France) were added as internal standards. Then purification was realized using acidified silica columns (Silica gel and Sulfuric acid, Sigma-aldrich) and ^13^C-labelled PCB 111 (Molsheim, France) was added as external standard. Finally quantification was achieved by a GC-MS (7 890A, Agilent Technologies, Santa Clara, USA). All chemicals used in this study were pesticide residue analytical grade. Details are provided in previous publication [17].

### 5 Data analyses

#### 5.1 Quality control of the data set

Regardless the treatment group, PCB 28 levels were beneath the limit of quantification and were not incorporated in the data set. Data from 2 piglets were excluded from analysis because these piglets presented digestive issues before and during the exposure part of the experiment (diarrhoea, low daily food intake and weight gain). These issues could affect PCB absorption. Two additional values for PCB 52 were lower than the limit of quantification.

#### 5.2 Effect of OM on NDL-PCBs’ adipose tissue concentrations

In order to assess the impact of the different matrices on bioavailability of the contaminant, variance analyses were performed. Concentrations of each NDL-PCB congener in adipose tissue were compared between the different treatment groups (n  = 4 to 5 for each group) using the GLM procedure and Tukey-Kramer *post-hoc* test in SAS (Statistical Analysis Software, SAS/STAT 9.1, SAS Institute Inc., Cary, USA). Differences were considered significant at p<0.05.

#### 5.3 Relative bioavailability (RBA) estimates calculation

In order to compare the bioavailability between the soils and the oil-reference, relative bioavailability was calculated as the ratio of the PCB concentration in adipose tissue from soil groups to those from the oil-group (100% reference), adapted from a method previously described [17], [18]. As adjusted means of concentrations of control animals did not differ from 0 (p>0.357) (see 2.5.2), data was not corrected with these values. In order to assess differences of retention by OM between congener, a multivariance analysis was performed. Statistical analyses were carried out using MIXED procedure SAS (9.1, SAS Institute) on RBA values. Each piglet was considered as an experimental unit. RBA values of each NDL-PCB congener in adipose tissue were analysed using mixed model with treatment group and NDL-PCB congeners as fixed factors and piglets as random one. The model included all treatment groups (n  = 4 to 5 for each group) and NDL-PCB congeners as effects. A Tukey-Kramer *post-hoc* test was used for comparison of means to detect differences between treatment groups. Differences were considered significant for p<0.05.

## Results

Daily ingestion of feed was unaffected by treatment during the exposure period (4.2±0.3% of BW.d^−1^, mean ± SD, P≥0.10). Moreover, no significant effect on daily weight gain (323±97 g.d^−1^, mean ± SD, P≥0.10) could be discerned at the end of the exposure period.

### 1 Effect of OM on NDL-PCBs concentrations in adipose tissue

**Figure 2:**
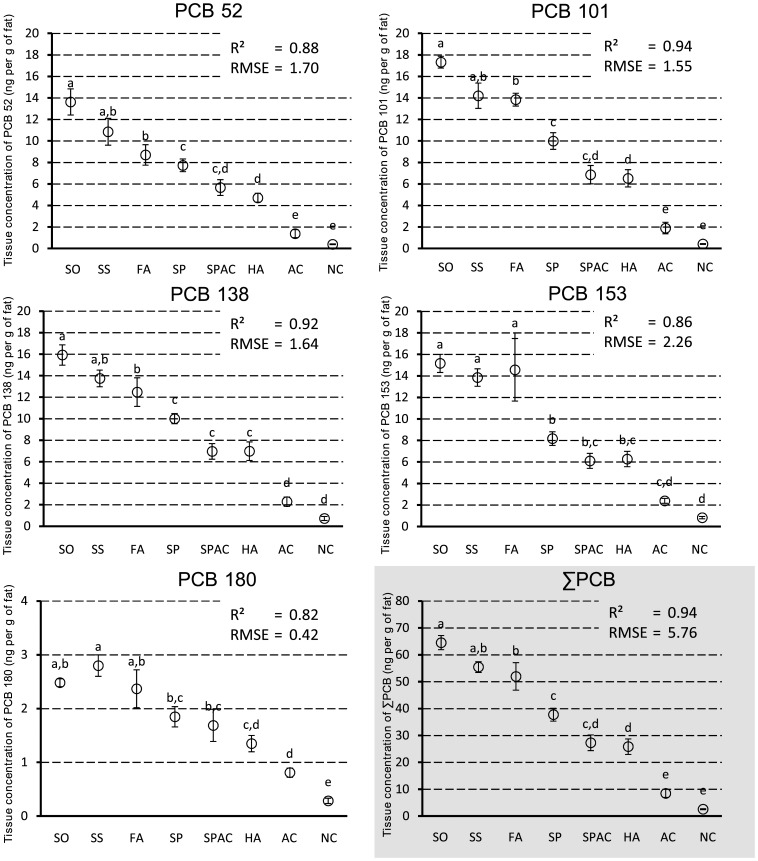
Concentrations (ng.g^−1^ of fat) of NDL-PCBs in adipose tissue. Each value represents the adjusted mean ± standard error. Values are presented following treatment groups: Spiked corn oil (SO, n  = 5); Standard soil without organic matter (SS, n  = 4); 1% OC Fulvic acid soil (FA, n  = 4); 1% OC *Sphagnum* peat soil (SP, n  = 5); 1% OC *Sphagnum* peat soil; Activated Carbon (95∶5) (SPAC, n  = 5); 1% OC Humic acid soil (HA, n  = 5); 1% Activated carbon (AC, n  = 5); Negative controls (NC, n  = 5). Letters (a, b, c, d, e, f) indicate values that do not statistically differ from other values within groups presenting a common letter (*P*<0.05).

Models of concentrations in adipose tissue for each congener were fitted with coefficients of determination ranging between 0.82 and 0.94 (*cf* Fig. 2). Focusing on the sum of detectable NDL-PCBs, 3 distinct sets of response to treatments were observed (*cf* Fig. 2 F) (n  = 4 to 5, p<0.05). In increasing order of concentration in adipose tissue, the first set comprises animals from NC and AC groups, the second one animals from HA, SP and SPAC groups, and the third one comprises animals from FA, SS and SO groups. All the sets were significantly different one from the other (p  = 0.018 for set 1 and 2; p<0.001 for set 1 and 3; p  = 0.001 for set 2 and 3). The first set exhibited very low concentrations of NDL-PCBs, with animals from the negative control group having the lowest ones (2.6±0.2 ng.g^−1^ of fat, mean±SE). Animals from the AC group presented slight but non-significant higher levels (8.5±1.5 ng.g^−1^ of fat, mean±SE; p  = 0.731). The second set comprises groups displaying intermediate levels of NDL-PCBs. Animals in the SPAC group presented lower concentrations than those in HA group and the SP one (respectively 25.8±2.9; 27,3±2.9 and 37.7±2.4 ng.g^−1^ of fat, mean±SE). HA group was not statistically different from the other ones within this set (p  = 0.114 for SPAC; p  = 0.999 for SP) whereas SP and SPAC groups were significantly different (p  = 0.048). At last, the third set exhibited the highest levels of NDL-PCBs and animals from SO groups exhibited higher concentrations than SS and FA ones (respectively 64.5±2.6, 55.4±2.0, 51.9±5.1 ng.g^−1^ of fat, mean±SE). In addition, SS group were not significantly different from the other one within this set (p  = 0.303 for FA; p  = 0.987 for SO) while SO and FA were significantly different (p  = 0.049). These observations can be roughly generalized to the separate analyses of almost all quantifiable NDL-PCBs congeners (PCB 52, PCB 101, PCB 138 and PCB 153) except for PCB 180. Due to lower exposure levels of PCB 180 compared to the other congeners and lowest concentrations in adipose tissue, higher variations were obtained with less differences between the treatment groups.

### 2 Impact of OM on RBA estimates

In order to assess differences in retention by OM between the 5 quantifiable NDL-PCBs, RBA factors were calculated (5 NDL-PCBs, n  = 4 to 5, *cf* Table 2). In general, RBA estimates from all soil groups were statistically lower than those from SO except for the SS group. In consequence, RBA estimates for SS and FA groups are statistically not different from 100%.

**Table 2:** Relative bioavailability (RBA) factors of NDL-PCBs.

	PCB 52		PCB 101		PCB 138		PCB 153		PCB 180		LSMEANS PCB	
Spiked corn oil (SO)	**100** ^a^	[88.4 −112]	**100** ^a^	[88.4 −112]	**100** ^a^	[88.4 −112]	**100** ^a^	[88.4 −112]	**100** ^a^	[88.4 −112]	**100** ^a^	[91.6 −109]
Standard soil (SS)	**79.7** ^a,b^	[66.6 −92.7]	**82.0** ^a,b^	[69.0 −95.0]	**86.3** ^a,b^	[73.3 −99.3]	**91.3** ^a^	[78.3 −104]	**113** ^a,b^	[99.8 −126]	**90.4** ^a^	[81.0 −99.9]
Fulvic acid (FA)	**63.9** ^b,c^	[50.8 −76.9]	**79.9** ^a,b^	[66.9 −92.9]	**78.3** ^a,b^	[65.3 −91.3]	**96.1** ^a^	[83.0 −109]	**95.5** ^a,b,c^	[82.5 −109]	**82.7** ^a^	[73.3 −92.2]
Sphagnum peat (SP)	**56.7** ^b,c^	[45.1 −68.4]	**57.7** ^b,c^	[46.0 −69.3]	**62.8** ^b,c^	[51.1 −74.4]	**53.9** ^b^	[42.3 −65.5]	**74.5** ^b,c,d^	[62.9 −86.2]	**61.1** ^b^	[52.7 −69.6]
Humic Acid (HA)	**41.6** ^c,d^	[30.0 −53.2]	**39.6** ^c,d^	[27.9 −51.2]	**43.7** ^c,d^	[32.0 −55.3]	**40.2** ^b,c^	[28.5 −51.8]	**68.0** ^c,d^	[56.4 −79.7]	**46.6** ^b,c^	[38.2 −55.1]
Sphagum peat andactivated carbon (SPAC)	**34.6** ^c,d,e^	[23.0 −46.3]	**37.6** ^c,d^	[26.0 −49.3]	**43.8** ^c,d^	[32.2 −55.5]	**41.4** ^b,c^	[29.7 −53.0]	**54.4** ^d,e^	[42.7 −66.0]	**42.4** ^c^	[33.9 −50.8]
Activated carbon (AC)	**9.6** ^d,e^	[−3.0 −22.3]	**11.0** ^d,e^	[−0.7 −22.6]	**14.3** ^d,e^	[2.7 −26.0]	**15.7** ^c,d^	[4.1 −27.4]	**32.6** ^e,f^	[21.0 −44.3]	**16.7** ^d^	[8.2 −25.1]
Negative controls (NC)	**3.3** ^e^	[−9.4 −15.9]	**2.4** ^e^	[−9.2 −14.1]	**4.5** ^de^	[−7.1 −16.2]	**5.4** ^d^	[−6.3 −17.0]	**11.4** ^f^	[−0.3 −23.0]	**5.4** ^d^	[−3.1 −13.9]
**LSMEANS MODEL PCB**	**48.7^C^**	[44.5 −52.9]	**51.3^C,B^**	[47.2 −55.3]	**54.2^C,B^**	[50.2 −58.3]	**55.5^B^**	[51.4 −59.5]	**68.7** ^A^	[64.6 −72.7]		
Residual	0.010	OM effect:	p<0.001									
RMSE	0.13	PCB effect:	p<0.001									
		PCB*OM:	p = 0.050									

RBA factors were calculated from adjusted means of concentrations (ng.g^−1^ of fat). Values in brackets indicates 95% confidence interval (2xSE). SE were calculated via propagation of errors formula.

Multivariate analysis was performed using GLM procedure on RBA values 95% confidence interval (2xSE) was calculated.

Organic matter effect: letters (a, b, c, d, e, f) indicate values that do not statistically differ from other values within column presenting a common letter (P<0.05).

NDL-PCBs effect: letters (A, B) indicate values that do not statistically differ from other values within line presenting a common letter (P<0.05).

RMSE: Root means square error.

Considering results of SP, SPAC and AC groups several assumptions could be realized looking at RBA estimates of the sum of congeners together (*cf* Table 2, last column). Firstly, SP phase significantly reduced RBA estimates compared to FA, but less than HA (p<0.001 for both). Secondly, a significant reduction of RBA estimates was achieved from the SP (61.1%, lsmean) to the SPAC group (42.4%, lsmean) (p<0.001). An even larger reduction of RBA estimates was observed comparing SP (61.1%, lsmean) and AC (16.7%, lsmean) groups (p<0.001). In addition, RBA estimates from AC group on its own were significantly lower (16.7%, lsmean) than the SPAC ones (42.4%, lsmean) (p<0.001). At last, all treatment groups were statistically different from the NC group except the AC one (p  = 0.571). These results lead to consider competition between the two organic phases SP and AC and as the consequence of this, a smaller reduction.

Preferential capacities of retention of each congener linked to OM was performed studying the interaction between both effects (OM and NDL-PCB congener) on RBA estimates (*cf* Table 2). Firstly, all data were used and both the effect of OM quality of the soils and of the NDL-PCB congener were found to be significantly different (p<0.001 for both). Analysing differences between NDL-PCB congeners showed a significant higher RBA estimate for PCB 180 (p<0.001) and those estimates increased along the chloration degree (*cf* Table 2, last line). These results may indicate an increase of deposit for the more chlorinated congeners regardless the OM and is in kept with the particular lipophilic properties of those ones.

## Discussion

**Figure 3:**
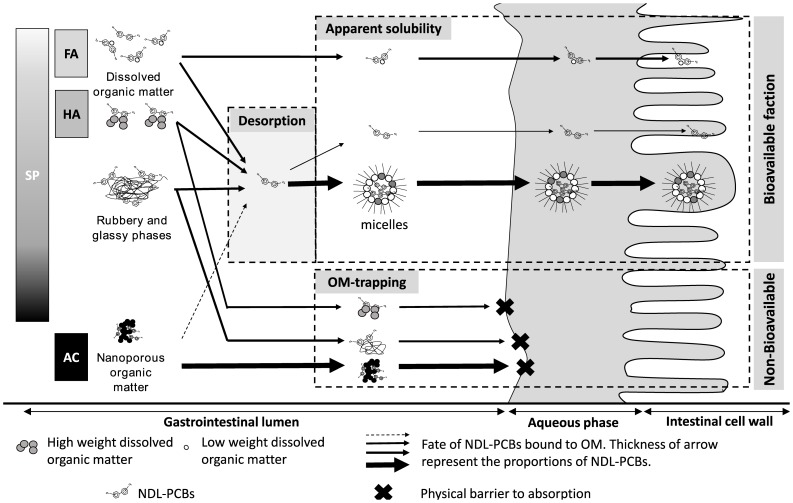
Mechanistic approach of retention and bioavailability of NDL-PCBs bound to OM.

It is known that NDL-PCBs in the intestinal lumen are highly absorbed and that the digestive processes in humans mainly contribute to enhance the absorption of such pollutants. Two main processes are believed to explain the high absorption efficiencies of lipophilic POPs [23]. They concern the apparent solubilisation process and the digestive fluxes against the mucosa supported by concentrations gradient [23], [24] (*cf* Fig. 3). Apparent solubility mainly results from lipidic micelles transportation in the intestinal lumen [23]. These both mechanisms also explain that lipophilic compounds could transfer to intestinal cells whereas they must cross successive aqueous phases (intestinal lumen, mucus phase, the unstirred water layer) [25], [26] (*cf* Fig. 3). Thus, properties of PCB as their log K_ow_ does not seem to have an important impact on efficiencies uptake as Humans present particularly high levels of absorption efficiencies of PCBs [24]. In contrast to the digestive processes which do not limit the absorption of organic pollutants, the results of this study clearly demonstrate that OM and its characteristics modulate considerably the bioavailability of NDL-PCBs sequestrated in soil.

In the present study we compared the absorption efficiency of a same amount of pollutants brought either in a reference media (oil) or in a tested one (soil with contrasted organic matter characteristics). As the oil is efficiently absorbed by young monogastric [27], as well as human [24], NDL-PCBs in the reference form are known to be highly bioavailable. Through the relative bioavailability methodology, results are normalised to this reference form to clearly determine if OM sequestrate efficiently NDL-PCBs along the digestive processes [17]–[20]. In consequence, a possible explanation of the results is that relative bioavailability for each single NDL-PCBs congener is mainly impacted by the extent of desorption of those PCBs bind to OM in the intestinal lumen. This desorption is assumed to be the limiting step, prior to apparent solubilisation.

Sequestration of POPs results from several weak physical interactions between them and OM. Several conceptual models have been set up to illustrate these binding properties of OMs, retention of POPs and desorption data in aqueous environment. The next steps of discussion will focus on two distinct model of physical strengths binding POPs to OM as described in the “glassy/rubbery” model and also in the “physical trapping” one.

The “glassy/rubbery” model for OM assumes that OM is divided in two different parts. The first one refers as “soft carbon” or “rubbery” supporting a partitioning domain and the second one called “hard carbon” or condensed “glassy” domain which provides a site-specific but extent-limited adsorption [28]. Xing and Pignatello describe a “fast fraction” of POPs that sorbed rapidly to OM describing the partitioning fraction of POPs to OM, whereas a “slow fraction” is continuously diffusing deeper within the OM, corresponding to a site-specific adsorption [29]–[31]. Both non-linear behaviour and hysteresis could be related to unequal retention of POPs by both OM phases [14], [28], [31]. This hysteresis materializes a recalcitrant fraction of POPs to diffuse and to solubilize, since it is bound to the glassy domain [29], [31]. Thus, for standard organic matter used in the present study, the “soft carbon” part should be predominant for FA, HA. In contrast, SP should have a more important “glassy” phase than FA and HA. One other possible effect of retention of POPs by soluble OM is that a transportation could occur. FA could also be absorbable and have a consistent impact on bioavailability of molecules [32], [33]. However it is assumed that their physical properties as their chemical functions or their length could be limiting factors. In the present study FA exhibits a low molecular weight (3 kDa to 16 kDa) [34] (*cf* Fig. 3). This element is also in accordance with the high bioavailability for the FA group in this study. In contrast, AC is known to strongly bind POPs, and this strong retention should be highlighted. Indeed, an even more efficient sequestration was secondarily described to take into account a nanoporous “physical trapping” of OC offered by solid particulate of condensed OM [14]. In the present study, OMs showed different K_oc_ ranging from 2.3 to 2.9 (respectively chosen FA and HA) whereas SP exhibited intermediate but more variable K_oc_ [35]. AC is known to tightly bound POPs and partitioning coefficient are fairly greater (above 6) [36]. RBAs found in this study (oil ≥ fulvic acid>*Sphagnum* peat ≥ *Sphagnum* peat and activated carbon ≥ Humic acid>>activated carbon) are in line with these K_oc_ values.

It also appeared that SPAC did not show low RBA levels as that found with AC. Studies dealing with competition between carbonaceous geosorbent and organic matter emphasize that nanopores of those condensed particles could be filled by adsorbed humic material. This adsorption leads for example to a lesser extent of PCB sorption [37] and could explain this specific result of the present study. In addition, PCB 180 is less retained by OM than the other NDL-PCBs. This observation appears controversial in regard to previous data published. Indeed K_oc_ is known to increase along the K_ow_; and as the highly chlorinated congeners are the most lipophilic ones this contradicts the present result [38]. As the sequestration of OCs in OM is a time-dependent process, the aging process could not be sufficient to allow correct integration of the highest congener. This aging procedure 1/was designed to be easily reproducible 2/to be sufficient to obtained a large differences of RBA responses.

In terms of risk assessment, the lowest levels of relative bioavailability were found in the ACs group. As AC is reported to be more nanoporous than black carbon [39], such decrease of relative bioavailability should not be expected in natural contaminated soils. Thus, in natural conditions, retention of NDL-PCBs by humic substances has been found to be limited as relative bioavailability values ranged from 39.3% to 96.1% (upper p95 value) for all soil groups except AC ones. Even if these results were obtained from an animal model, it could be considered that children are as efficient as piglets to desorb NDL-PCBs bound to OM. Therefore, in the context of involuntary ingestion of contaminated soil by children, it would be preferable to consider that the ingested dose correspond to the exposure dose.

## Conclusion

Humic substances impact differently the retention of PCBs during the digestive process in piglets. Indeed, a wide range of RBAs was achieved between all the prepared artificial soils. While AC (0.05%) strongly reduces bioavailability, competition with SP shows an important decrease of the retention. Commercial HA also appears to be an efficient media of retention. These elements are of great interest for further investigations in terms of risk assessment in contaminated areas in the context of involuntary soil ingestion by toddlers.
